# A case of bilateral amyloidosis localized to extraocular muscles mimicking thyroid eye disease

**DOI:** 10.1186/s12886-024-03295-y

**Published:** 2024-01-26

**Authors:** Natsuyo Yoshida-Hata, Masashi Mimura, Miwa Aikawa, Tomoyuki Kashima

**Affiliations:** 1 Oculofacial Clinic Tokyo, 1-15-4 Ginza, Chuo-ku, 104-0061 Tokyo, Japan; 2 Oculofacial Clinic Osaka, 1-12-6, Umeda Kita-ku, Osaka-city, 530-0001 Osaka, Japan

**Keywords:** Extraocular muscle amyloidosis, Thyroid eye disease, Proptosis, Diplopia

## Abstract

**Background:**

Amyloidosis is a rare condition characterized by the abnormal deposition of amyloid proteins in various tissues and organs. While systemic amyloidosis has been well-documented, amyloid deposition in extraocular muscles is an exceptionally rare occurrence, with only 35 reported cases. This case report sheds light on the importance of considering amyloidosis in the differential diagnosis of patients presenting with proptosis and diplopia, which are often associated with thyroid eye disease.

**Case presentation:**

A woman in her twenties sought medical attention due to a complaint of diplopia. Her ocular examination revealed almost normal findings except for exotropia and proptosis. Orbital magnetic resonance imaging displayed fusiform enlargement of nearly all eye muscles, a presentation typically observed in thyroid eye disease. However, despite corticosteroid therapy, her symptoms showed no improvement. Given the unusual lack of response to conventional treatment, and inhomogeneous enhancement of the muscle, an extraocular muscle biopsy was conducted. This biopsy yielded a unique finding—amyloid deposition within the muscle tissue. This discovery was particularly intriguing due to the extreme rarity of amyloidosis affecting extraocular muscles, with fewer than three dozen documented cases worldwide.

**Conclusion:**

This unique case underscores the critical need for a comprehensive approach to diagnosing patients with proptosis and diplopia. While these symptoms are commonly attributed to thyroid eye disease, it is essential to consider alternative diagnoses such as amyloidosis, especially when standard treatments fail to yield results. The discovery of amyloid deposition in the extraocular muscles, although exceedingly rare, emphasizes the significance of a thorough differential diagnosis. In conclusion, this case report highlights the importance of vigilance in clinical practice, encouraging ophthalmologists to explore less common diagnostic possibilities when faced with challenging cases. Further research and clinical investigation are warranted to better understand the mechanisms and potential treatments for amyloidosis affecting the extraocular muscles.

## Background

Amyloidosis is a rare disease characterized by the accumulation of abnormal amyloid fibrillar protein, in various tissues [1, 2]. Ocular amyloidosis has been documented in nearly all parts of the eye, including adnexal and orbital tissues [2]. Nonetheless, involvement of the extraocular muscles is an exceedingly rare [1–3]. To date, a mere 35 cases of localized amyloidosis in the extraocular muscles, with or without systemic involvement, have been reported in the literature [1–15]. The diagnostic process can be intricate due to the resemblance of clinical features to thyroid eye disease [4]. In this study, we present a case of isolated amyloidosis initially misdiagnosed as thyroid eye disease, and we delve into the diagnostic challenges encountered.

## Case presentation

**Fig. 1:**
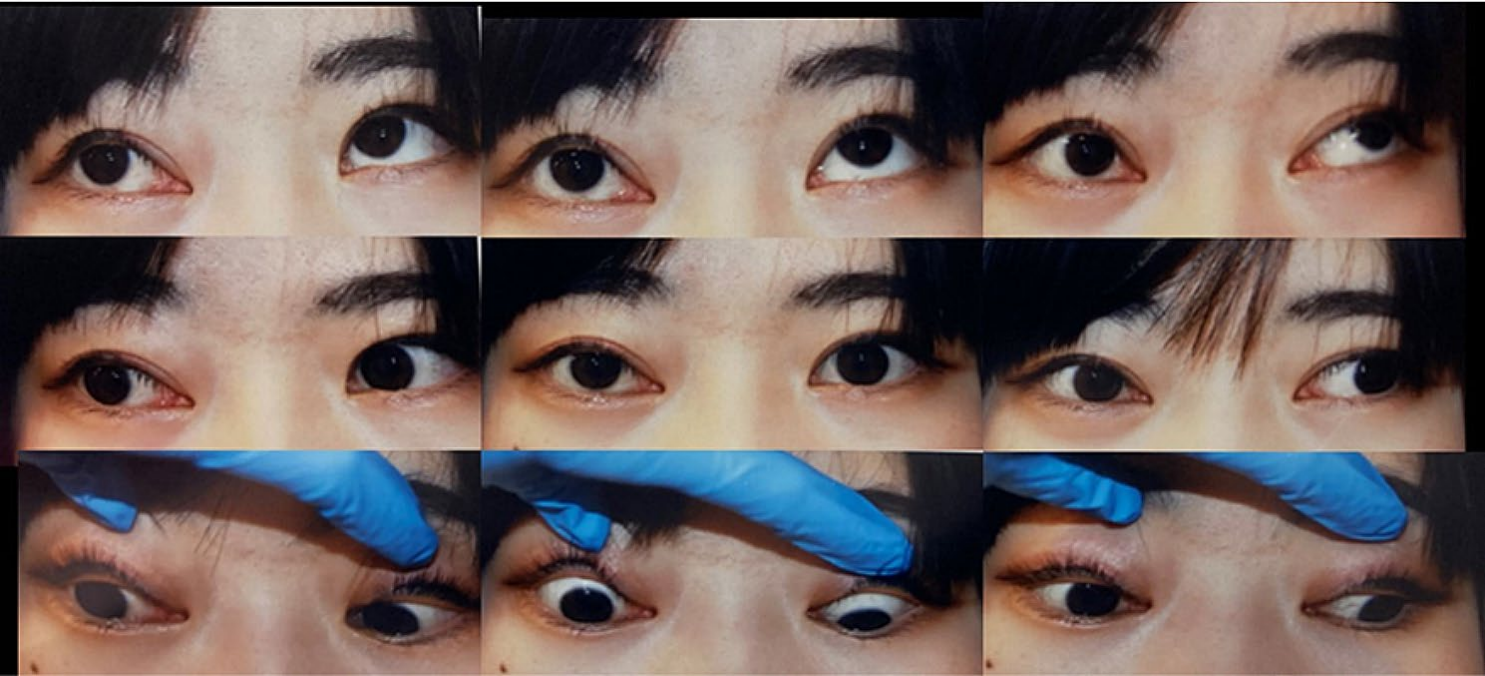
Nine diagnostic gaze position photographs of the patient illustrating limitation of right eye movement in all gaze directions. The right eye exhibits greater proptosis and eyelid swelling, although no lid lag was observed

A 24-year-old woman was referred to us, presenting a five-year history of diplopia in left gaze. Prior to her referral, she had been treated for diplopia in the left gaze, with the previous doctor suspecting the thyroid eye disease as the cause. Her overall health was good with no past medical history including ophthalmology examinations. The examination revealed exotropia and slight right hypertropia, as follows; in the Alternate Prism Cover Test (APCT), there was 10 prism diopters of exotropia and 2 prism diopters right hypertropia, attributed to limited ocular motility (Fig. 1). The degree of limitation was − 2 for inferior oblique muscle and lateral muscle, and − 3 for the superior rectus, inferior rectus and superior oblique muscle. The patient’s visual acuity was 20/20 in both eyes, and her pupils were round, yet the right pupil appeared tonic and dilated. Critical fusion frequency (CFF) and visual field tests were within normal limits. Anterior segment and fundus examinations yielded unremarkable findings. Proptosis of both eyes, with the right eye predominant were evident, resulting in a 3 mm difference in proptosis between the eyes. However, her upper eyelids were not retracted.

**Fig. 2:**
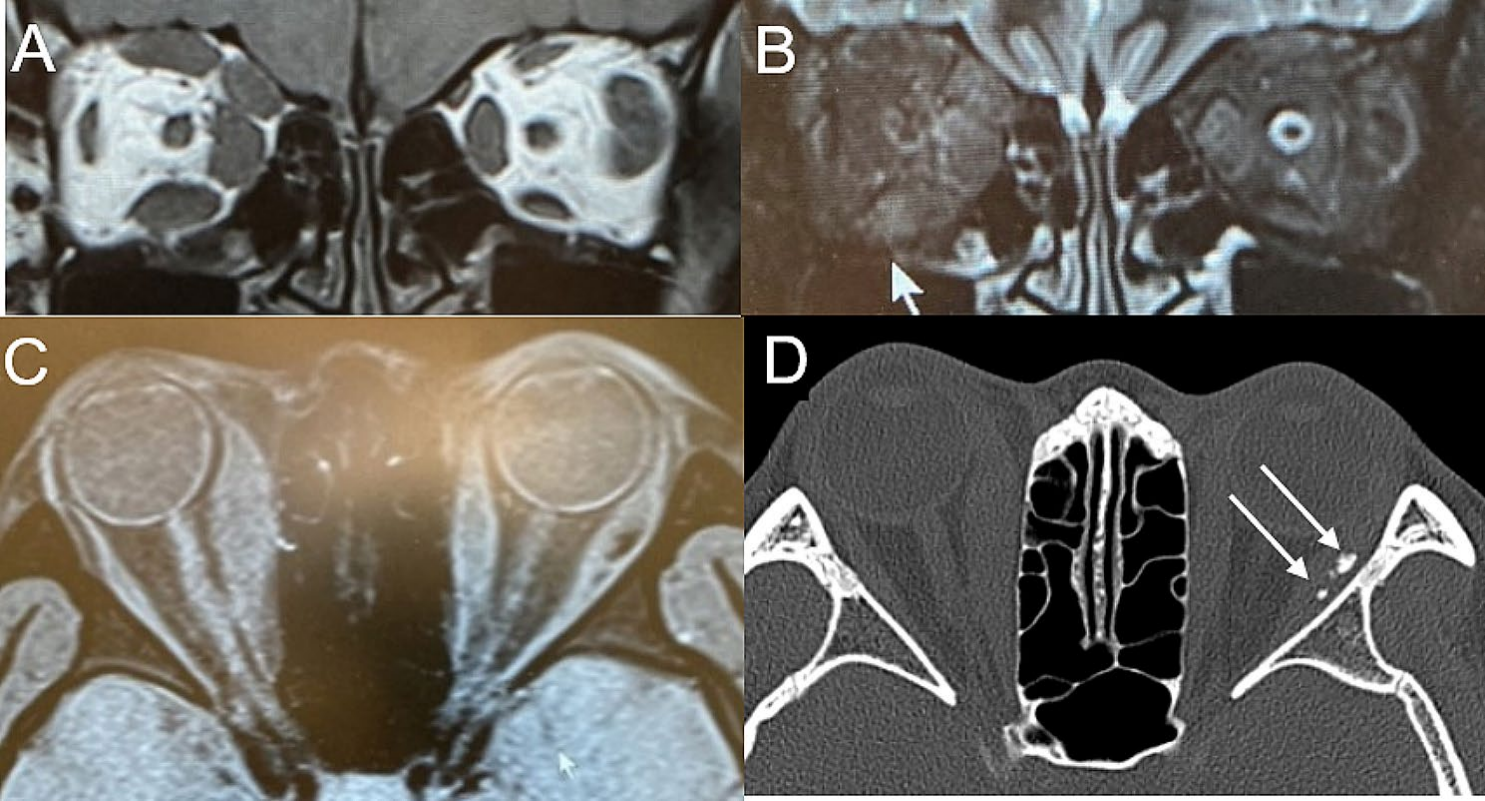
(**A**) Coronal T1-weighted MRI image depicting bilateral extraocular muscle thickening with tendon sparing (**B**) Coronal orbital image using Short-Tau Inversion-Recovery (STIR) sequence, highlighting inhomogeneous enhancement in the external muscles of both eyes (**C**) Axial T1-weighted MRI image displaying enlargement of bilateral medial rectus muscles and left lateral rectus (**D**) CT scan revealing calcification (arrow) within the right lateral rectus muscle

Computed tomography (CT) and magnetic resonance imaging (MRI) unveiled fusiform enlargement of nearly all muscles in the right eye except the lateral rectus muscle and lateral and medial rectus in the left eye (Fig. 2a-c). Additionally, CT revealed calcification within the right lateral muscle (Fig. 2d). The enlarged muscles displayed slightly heightened signal intensity on short-tau inversion-recovery (STIR) MRI, accompanied by irregularly enhanced areas. Laboratory investigations indicated a slightly reduced thyroid-stimulating hormone (TSH) level (0.381 IU/ml), along with slightly elevated anti-thyroid peroxidase antibodies. However, levels of free triiodothyronine, free thyroxine, and TSH receptor antibodies were within the normal range. Furthermore, levels of serum C-reactive protein, IgG4, soluble interleukin-2 receptor, angiotensin-converting enzyme, and lysosomal enzymes were all within normal limits.

**Fig. 3:**
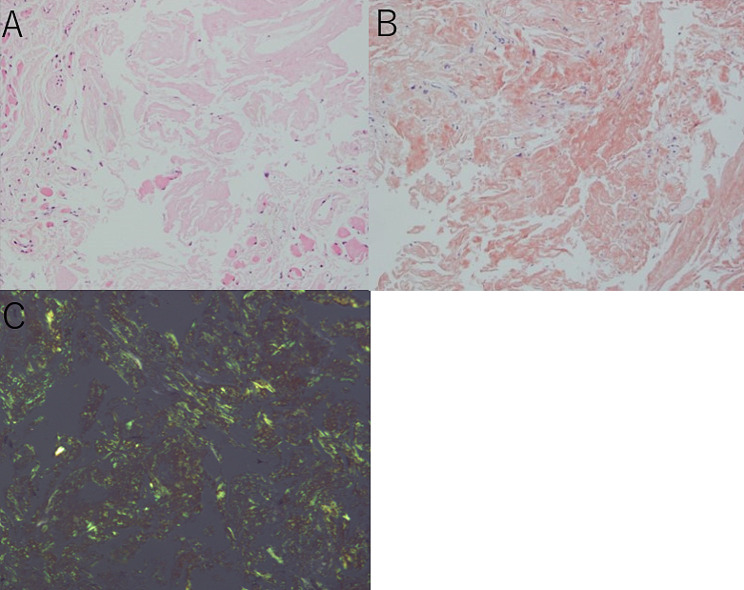
Histopathological examination of the right medial rectus muscle: (**A**) Hematoxylin and eosin staining at ×200 magnification, revealing widespread eosinophilic amorphous material amidst atrophied muscle fibers (**B**) Direct fast scarlet stain at ×200 magnification, indicating positive staining of the eosinophilic material (**C**) Polarized light microscopy showing apple-green birefringence of the material

With the exception of the absence of upper eyelid retraction and the presence of calcification in the extraocular muscle, clinical features were consistent with thyroid eye disease (TED). Consequently, a postbulbar triamcinolone injection was administered to mitigate the risk of compressive optic neuropathy. However, this intervention yielded no improvement, prompting consideration of alternative diagnoses. Subsequently, a biopsy of the right medial rectus muscle was performed via a conjunctival incision near the semilunar fold. The muscle, measuring 5 mm in width and 9 mm in length, appeared fragile and exhibited a yellowish hue. Histopathological evaluation demonstrated muscle fiber atrophy and focal loss, accompanied by widespread deposition of eosinophilic amorphous material visible through hematoxylin and eosin staining. Amyloid-specific birefringence upon polarized microscopy and positive direct fast scarlet staining confirmed the presence of orbital amyloidosis (Fig. 3). Amyloidosis subtypes was A-type amyloidosis, but further detail subtype was not investigated. Comprehensive systemic evaluations, including cerebral MRI, plasma and urine protein electrophoresis, abdominal ultrasound, echocardiography, and bone marrow biopsy, revealed no evidence of systemic amyloidosis. Thus, a diagnosis of primary isolated amyloidosis of the extraocular muscle was established.

Over an 18-month follow-up period post-biopsy, there were no changes in visual acuity or ocular motility. Moreover, no indications of systemic amyloidosis emerged, obviating the need for systemic interventions such as high-dose melphalan and autologous stem cell transplant.

## Discussion and conclusions

This case report showcases a rare instance of primary localized amyloidosis in the extraocular muscle mimicking thyroid eye disease. The combination of MRI and CT findings, unresponsiveness to conventional steroid therapy, and muscular biopsy revealed the presence of amyloid deposition, confirming the diagnosis.

Notably rare, orbital amyloidosis is documented in 0.3% of orbital disease cases [1, 16]. Instances of extraocular muscle amyloidosis, even rarer, have only been reported in 35 cases with and without systemic involvement [1–15]. Clinical differentiation between extraocular muscle amyloidosis and thyroid eye disease is intricate due to shared features [4, 17]. Manifestations of extraocular muscle amyloidosis include painless proptosis and diplopia resulting from external ophthalmoplegia, often progressive, mirroring those seen in thyroid eye disease. The two conditions are also challenging to differentiate through imaging, as both may lead to fusiform muscle enlargement. Amyloid deposition typically exhibits either hypointense or isointense signals on T1 sequences and sometimes appears hypointense on T2 sequences [7]. Notably, inhomogeneous enhancement on MRI and calcification on CT emerge as distinct features [4, 7, 8].

Several conditions, such as thyroid eye disease, orbital inflammation, carotid cavernous fistulae, metastatic tumors, and IgG4-related disease, may underlie enlarged extraocular muscles (Table 1) [17]. Calcified orbital lesions are evident in various conditions, including metastatic orbital tumors, meningioma, teratoma, neurofibroma, sclerosing hemangioma, and amyloidosis. However, clinical findings and laboratory data facilitate differentiation from amyloidosis. For instance, metastatic tumors can be distinguished through medical history and MRI findings [17].

**Table 1:** Diffrential diagnosis

Differential diagnosis	Symptoms	Steroid reactive	CT finding	MRI findings
Amyloidosis	Diplopia, ophthalmoplegia, proptosis	-	Fusiform muscle enlargement, calcification, adjacent hyperostosis, bony irregularity	Fusiform muscle enlargement, heterogenous compositiom, T1and T2: not specified signal intensity
Thyroid eye disease	Diplopia, ophthalmoplegia, proptosis, eyelid retraction, lid lag, red eye, optic neuropathy, lid swelling, and/or periorbital swelling	+	Fusiform muscle enlargement, tendinous insertion is less commonly involved	Fusiform muscle enlargement with sharp borders, T1: isointense to the other facial muscles, or fatty infiltration, T2: high, STIR: high signal intensity
Metastatic tumor	Ophthalmoplegia, proptosis, with or without diplopia	-	Variable, soft tissue attenuating material	T1: isointense to muscle, T2: hyperintense to muscle, hypointense to fat
IgG4 related disease	Lacrimal gland involvement, painless, periorbital and/or lid swelling, erythema, proptosis, with or without diplopia	+	Enlargement of the lacrimal gland and/or extraocular muscle(s)	T1: isointence to muscle, T2: hyperintence to muscle
Carotid cavernous fistulae	Conjunctival injection, pulsatile proptosis, diplopia	-	Dilation of the superior ophthalmic vein	dilation of the supreior ophthalmic vein
Idiopathic orbital inflammation	Periorbital edema, swelling, ophthalmoplegia, rapid-onset, usually unilateral, painful proptosis and diplopia	+	Enlargement of the muscle belly typically with the involvement of tendinous insertions	T1: typically isointence or hypointence, T2: typically hypointense

Legends: Differential diagnosis of extraocular muscle amyloidosis. STIR: short tau inversion recovery

Following confirmation of ocular amyloid deposition, a thorough investigation for systemic amyloidosis is imperative. Previous reports indicate that approximately 50% of patients with isolated amyloid deposition in extraocular muscles are found to have systemic amyloidosis [2]. Fortunately, our patient displayed no systemic amyloidosis over an 18-month period. Continuous follow-up remains essential to detect potential onset of systemic amyloidosis.

We present an exceptional case of primary isolated amyloidosis in the extraocular muscle, masquerading as thyroid eye disease. This case underscores the possibility of atypical extraocular muscle enlargement being attributed to amyloidosis, thus advocating for tissue biopsy when conventional therapies yield no results. Notably, prompt diagnosis of ocular amyloidosis necessitates a systemic work-up, given the potential life-threatening systemic involvement.

## Data Availability

Not applicable.

## Abbreviations

CFF: Critical fusion frequency
CT: Computed tomography
MRI: Magnetic resonance imaging
STIR: Short-tau inversion-recovery
TSH: thyroid-stimulating hormone
TED: Thyroid eye disease
